# Encapsulation of Olive Pomace Extract in Rocket Seed Gum and Chia Seed Gum Nanoparticles: Characterization, Antioxidant Activity and Oxidative Stability

**DOI:** 10.3390/foods10081735

**Published:** 2021-07-27

**Authors:** Alican Akcicek, Fatih Bozkurt, Cansu Akgül, Salih Karasu

**Affiliations:** 1 Department of Food Engineering, Davutpasa Campus, Faculty of Chemical and Metallurgical Engineering, Yildiz Technical University, Istanbul 34210, Turkey; akcicekalican82@gmail.com (A.A.); fh.bozkurt@gmail.com (F.B.); cansuakgul@aydin.edu.tr (C.A.); 2 Department of Gastronomy and Culinary Arts, Faculty of Tourism, Kocaeli University, Kocaeli 41080, Turkey; 3 Department of Food Engineering, Engineering and Architecture Faculty, Muş Alparslan University, Muş 49250, Turkey; 4 Food Quality Control and Analysis Program, Anadolu BİL Vocational High School, Istanbul Aydın University, Istanbul 34295, Turkey

**Keywords:** rocket seed gum, chia seed gum, olive pomace, encapsulation, nanoparticle

## Abstract

The aim of this study was to determine the potential use of rocket seed and chia seed gum as wall materials, to encapsulate and to prevent degradation of olive pomace extract (OPE) in polymeric nanoparticles, and to characterize olive pomace extract-loaded rocket seed gum nanoparticles (RSGNPs) and chia seed gum nanoparticles (CSGNPs). The phenolic profile of olive pomace extract and physicochemical properties of olive pomace, rocket seed gum (RSG), and chia seed gum (CSG) were determined. The characterization of the nanoparticles was performed using particle size and zeta potential measurement, differential scanning calorimeter (DSC), Fourier-transform infrared spectroscopy (FTIR), scanning electron microscope (SEM), encapsulation efficiency (EE%), in vitro release, and antioxidant activity analyses. Nanoparticles were used to form oil in water Pickering emulsions and were evaluated by oxitest. The RSGNPs and CSGNPs showed spherical shape in irregular form, had an average size 318 ± 3.11 nm and 490 ± 8.67 nm, and zeta potential values of −22.6 ± 1.23 and −29.9 ± 2.57, 25 respectively. The encapsulation efficiency of the RSGNPs and CSGNPs were found to be 67.01 ± 4.29% and 82.86 ± 4.13%, respectively. The OPE-RSGNP and OPE-CSGNP presented peaks at the 1248 cm^−1^ and 1350 cm^−1^ which represented that C-O groups and deformation of OH, respectively, shifted compared to the OPE (1252.53 cm^−1^ and 1394.69 cm^−1^). The shift in wave numbers showed interactions of a phenolic compound of OPE within the RSG and CSG, respectively. In vitro release study showed that the encapsulation of OPE in RSGNPs and CSGNPs led to a delay of the OPE released in physiological pH. The total phenolic content and antioxidant capacity of RSGNPs and CSGNPs increased when the OPE-loaded RSGNPs and CSGNPs were formed. The encapsulation of OPE in RSGNPs and CSGNPs and the IP values of the oil in water Pickering emulsions containing OPE-RSGNPs and OPE-CSGNPs were higher than OPE, proving that OPE-loaded RSGNPs and CSGNPs significantly increased oxidative stability of Pickering emulsions. These results suggest that the RSG and CSG could have the potential to be utilized as wall materials for nanoencapsulation and prevent degradation of cold-pressed olive pomace phenolic extract.

## 1. Introduction

Olive pomace is a low-cost and heterogenous solid waste that is obtained from olive oil production. Olive pomace could be considered a renewable source of high added value compounds, such as polyphenols [1] and they can be used in the food industry as an antioxidant source [2,3]. Olive pomace contains many phenolic compounds with potential influence on human health, such as hydroxytyrosol, tyrosol, and luteolin [4]. The primary phenolic compounds of olive pomace are oleuropein (1.22–13.50 mg/g), hydroxytyrosol (0.61–8.70 mg/g), tyrosol (0.13–1.115 mg/g), luteolin (0.02–0.14 mg/g), vanillin (0.92–3.64 mg/g), and rutin (0.21–1.70 mg/g) [5,6]. However, phenolic compounds have poor stability, low solubility, low bioavailability and are easily decomposed when exposed to both environmental (light, oxygen, temperature, humidity) and gastrointestinal (pH, digestive enzymes) system conditions. Efficient delivery systems should be designed to slow or prevent the degradation of the olive pomace phenolic compounds. Nanoencapsulation of phenolic compounds could be an efficient technique to prevent phenolic degradation and protects against harsh environmental and gastrointestinal system conditions. Nanoencapsulation has some advantages such as improving solubility and bioavailability, increasing stability, controlled release, valuable fortification of foods, and masked unpleasant taste [7,8]. Furthermore, nanoencapsulation of bioactive compounds can improve their efficiency, specificity, and targeting ability [9]. Nanoencapsulation techniques have some disadvantages, such as particle growth control, using organic solvents, being difficult to scale up, need for purification, and low encapsulation efficiency [10].

Nanoparticles are described as submicron-sized solid carriers which could be used for nanoencapsulation of bioactive compounds. Depending on the preparation method of the nanoparticles, they can be obtained as two different forms: nanospheres or nanocapsules. Nanospheres are matrix systems where the bioactive compounds are uniformly dispersed in the particles or absorb them at their surface. Nanocapsules have a vesicular system with a central core surrounded by a polymeric membrane and the bioactive compounds may dissolve into the inner core or adsorb onto the capsule surface [11]. Biodegradable nanoparticle systems have gained interest in recent years due to their potential delivery of susceptible bioactive compounds [7,12]. Nanoparticle systems could improve bioavailability drug-controlled release and soluble bioactive compounds compared to microparticles [13]. The reduced size of particles leads to a significant increase in surface to volume ratio, which ensures different physical–chemical and biological properties such as higher solubility and bioactivity, increased stability and cellular uptake, improved bioavailability, chemical reactivity, and controlled release of active compound [14,15]. Nanoparticle systems especially provide a polymeric barrier against harsh environmental conditions such as oxygen, light, and temperature [16]. Bioactive compounds are sensitive to pH, light, oxygen, and heat resulting in low bioavailability and bioactivity. However, the polymeric barrier protects bioactive compounds against oxidation under acidic and alkaline conditions in the stomach and the small intestine, respectively [17]. Food biopolymeric nanoparticles with these properties are currently being explored and have gained much attention in recent years [18]. However, polysaccharide-based biopolymeric nanoparticle systems have some disadvantages such as origin of the natural material, polydispersity and molecular weight controlling complications, resulting in batch-to-batch variability in the nanoparticles [19].

Emulsification, desolvation, and coacervation methods are the common techniques used for production of polymeric nanoparticles from polysaccharides and proteins [14]. Among the nanoparticle production methods, desolvation is an effective technique to produce nanoparticles due to low cost and easy operation [11,14]. Desolvation is a thermodynamically driven, self-assembly process with slow addition of different desolvating agents such as salts, alcohols, or solvents in a solution of macromolecules to regulate the precipitation of the polymers in the aqueous phase. The biopolymer concentration, pH, and amount of desolvation agent are important parameters for the physicochemical properties of nanoparticles [9,14].

Biopolymeric nanoparticles could be fabricated by using natural or synthetic polymers as wall material. Natural hydrophilic biopolymers such as gums and mucilage play an essential role in the fabrication of nanoparticles due to having properties such as availability, potential biodegradability, and high hydration capacity. These polymers, primarily found in plants, have highly water-soluble polysaccharides. Competing with synthetic polymers, natural hydrophilic polymers are considered ideal materials due to their low cost, non-toxicity, and the fact that they could be chemically modified [12,20].

Rocket seed is a plant from the Brassicaceae family, found in India and Southern Europe. Rocket seed epidermal cells are comprised of mucilaginous materials [21]. Rocket seed gum (RSG) is an anionic polysaccharide and, due to having mucilaginous materials, are promising polymers as wall materials. Chia seed is a common plant in Southern Mexico that belongs to the Lamiaceae family. Parts of the chia seed, such as flour, whole seeds, seed oil, and mucilage, could be useful in the food industry [22]. Chia seed gum (CSG) is an anionic polysaccharide and a good source of fiber and water hydration capacity. Due to its characteristics, CSG is a promising polymer to produce biopolymeric nanoparticles [23].

RSG and CSG are natural hydrophilic biopolymers and could have the potential to be utilized as wall materials. Many studies have been developed and evaluated for olive pomace and olive leaf phenolics encapsulation with different wall materials such as chitosan, maltodextrin, whey protein concentrate, and polylactic acid [1,24,25,26,27]. Nevertheless, there are no reports about olive pomace encapsulation by using RSG and CSG as wall materials. Firstly, this study aimed to determine the potential utilization of RSG and CSG as wall materials, prevent degradation of olive pomace phenolic extract by nanoencapsulation in polymeric nanoparticles, and characterize olive pomace extract-loaded RSG and CSGNPs.

## 2. Materials and Methods

In this study, olive pomace (Ekin Kocadag Olive and Food Industry) was provided and used as the main raw material. Olive pomace was obtained by the production of extra virgin olive oil with a 3-phase system. Olive pomace was dried at 50 °C for 7 h in a tray dryer (Intermak, Milkol, Turkey) and kernels being removed by sieving then ground with a flour mill type grinder (Karmatte Flour Mill, Meltas Machine, Turkey). Olive pomace powder (OPP) was stored in an airtight plastic container until analysis. RSG and CSG extracted from rocket and chia seeds were obtained from local producers. Tween 20 and ethanol were purchased from Sigma Aldrich. All the other reagents utilized were of analytical grade.

### 2.1. Extractions of Rocket Seed and Chia Seed Gum

Gums were obtained from rocket and chia seeds according to a modified method described by Razavi et al. (2009) [28]. A total of 1 lt distilled water was added to 50 g of seed and stirred on a magnetic heater (Heidolph MR3001) at 80 °C for 2 h. The solution was then filtered to separate the seeds, and 500 mL water in the solution was evaporated and mixed with ethanol (96%) in a ratio of 1:2 (water:ethanol) and the mixture was left overnight at 4 °C to allow gum to accumulate on the solution surface. Then, collected gum from the surface was dried in an oven (50 °C) for 1 day, and dried RSG and CSG were obtained.

### 2.2. Proximate Analysis of Olive Pomace Powder, Rocket and Chia Seed Gum

The protein, ash, moisture, fat content of olive pomace powder, RSG, and CSG were determined according to the standard Association of Official Analytical Chemist methods [29]. The Kjeldahl method was used to determine the total protein content, and the correction factor utilized was 6.25. The ash content was determined in a muffle furnace set to 550 °C. The moisture content was determined by gravimetry, maintaining the sample at 105 °C in an oven (Memmert UF 110. Germany) until constant weights were obtained. A Soxhlet extractor (Buchi E-216, Switzerland) was utilized to analyze the fat content. The carbohydrate content of RSG, CSG, and OPP was calculated by difference. The results were given in % *w*/*w* percentage.

Gum concentrations in the range of 0.1–2% *w*:*v* were used to determine the intrinsic viscosity. Using the following equations (Equations (1) and (2)), relative viscosity (*η_rel_*) and specific viscosity (*η_sp_*) were calculated:

(1) $$n_{rel}=\frac{n}{n_{s}}$$

(2) $$n_{sp}=\frac{n-n_{s}}{n_{s}}$$

where *η* is apparent viscosity of RSG, CSG and $n_{s}$ is viscosity of solvent [30]. Intrinsic viscosity ([*η*]) was defined by extrapolation of *η_sp_*/*C* to zero concentration using Huggins equation (Equation (3)) [30].

(3) $$\frac{n_{sp}}{C}=\left[n\right]+k_{H}{\left[n\right]}^{2}C$$

where *η*_sp_, *C*, [*η*], *k*_H_, are the specific viscosity (dimensionless), concentration of polymer (g dL^−1^), intrinsic viscosity (dL g^−1^), Huggins’ constant (dimensionless), respectively. A straight line is obtained when reduced viscosity or inherent viscosity is plotted against concentration according to Equation (3), and the intercept of this straight line provides the intrinsic viscosity value [31].

The Mark–Houwink–Sakurada (MHS) equation provides an indirect measure of molecular weight from intrinsic viscosity data:

(4) $$\left[n\right]=kM^{a}$$

where *n* is the intrinsic viscosity obtained from the Huggins’ equation (dL g^−1^), *k* is a constant (dL g^−1^), *M* is weight-average molecular weight (Da) and a is a function of polymer geometry (shaper factor) that is dimensionless [31].

The values of k and a vary with polymer conformation and the solvent–polymer system and are generally taken from the literature. Once *k* and a are known for a particular polymer and solvent–polymer system, one may use the intrinsic viscosity to determine the average molar weight of a polymer or vice versa. The k and a value of CSG were available in the literature and were determined by Timilsena et al. (2015) [31]. However, no literature data were available for RSG. Therefore, a modified Mark–Houwink equation was used to determine the molecular weight of the RSG [32]. A Mannose/Galactose ratio of the RSG was found to be 1.52 in a previous study [33].

(5) $$\left[n\right]=11.55\times 10^{-6}{\left[\left(1-X\right)Mv\right]}^{0.98}$$

where *M_v_* is the viscosity average molecular weight while $X=\frac{1}{\left[\left(\frac{M}{G}\right)+1\right]}$ and *M*/*G* is the mannose/galactose ratio of gum.

### 2.3. Preparations of Olive Pomace Extract

Before the extraction, olive pomace powder was defatted 3 times by using hexane. Olive pomace powder (80% methanol/water) was prepared in 100 mL and stirred (M5 CAT, Germany) at 600 rpm for 30 min. Then, it was mixed in a shaker mixer (Yamato Shaker MK200D, Japan) at 150 rpm for 90 min and centrifuged at 4500 rpm for 5 min to remove impurities. Then, the extract was filtered through filter paper (Whatman No. 4). All of the methanol was evaporated by a rotary evaporator (Buchi R-210, Switzerland). The extract was frozen for 24 h at −80 °C (EscoLexicon II Ult, Singapore), then dried in a freeze dryer (Christ Beta 1-8 LSC plus, Germany) and obtained in powder form.

### 2.4. Phenolic Compounds of OPE by HPLC Analysis

Phenolic profiles of OPE were determined by HPLC coupled to a diode array (HPLC–DAD). Standard calibration curves were prepared by using gallic, protocatechuic acid, catechin, p-hydroxybenzoic, syringic, elagic, m-coumaric, o-coumaric, myricetin, quercetin, kaempferol, hydroxytyrosol, tyrosol, and luteolin. The HPLC analysis was performed using a modified method [5]. The samples and stock solutions were filtered through a 0.45 µm membrane filter and analyzed in a Shimadzu HPLC system (LC-10AD vp pump, SPDM10A vp DAD detector, SIL-10AD vp autosampler, CTO-10AVP column oven, DGU-14A degasser, and SCL-10A system controller; Shimadzu Corp., Kyoto, Japan). Separations were performed at 30 °C on Agilent Eclipse XDB-C18 reversed-phase column (250 mm × 4.6 mm length, 5 μm particle size). The mobile phase contained solvent A (3% (*v*/*v*) acetic acid) and solvent B (methanol). A gradient elution was carried out as shown: 28% B (0–20 min), 28–30% B (21–50 min), 31–50% B (51–70 min), and 50–100% B (70–81 min) and at 90 min was returned to initial conditions. The flow rate was 0.8 mL/min. Chromatograms were recorded at 278 nm. Identification and quantitative analysis were made based on the retention times and external standard curves. The amounts of polyphenols were stated in μg/g of dried olive pomace extract.

### 2.5. Preparation of RSG and CSG Solutions

Next, 0.1% (*w*/*v*) gum was dissolved in distilled water for 2 h at 500 rpm at room temperature. Then, the gum solutions were left overnight to complete hydration at 4 °C. The gum was completely dissolved and then was centrifuged (Universal 320R, Germany) at 5000 rpm for 5 min to remove impurities. The pH values of gum solutions were adjusted to the 8 and 7 for RSG and CSG, respectively (HI 2211, UK) by using 0.1 N NaOH. The gum solutions were ready for further analysis.

### 2.6. Fabrication of RSG and CSG Nanoparticles

Gum nanoparticles containing OPE were produced by modified methods [14]. The prepared gum solutions (solvent) were mixed at 800 rpm for 5 min. OPE (0.1%) and Tween 20 (0.5%) was dissolved in optimized amounts of ethanol (Antisolvent). Tween 20 was used for better dissolution of OPE in ethanol. Ethanolic OPE (0.5 mL/min) was added dropwise to the gum solution (Solvent Phase) using a syringe pump system (New Era, NE, USA). After adding the organic phase, the solution was stirred at 800 rpm for 10 min. Then, by using ultrasonic processor (Hielscher UIP1000hdT, Germany), ultrasonication of 100 W was applied to the solutions for 1 min (every 30 s wait 10 s) in an ice bath. The remained nanoparticle suspensions were centrifuged at 9000 rpm for 30 min and the supernatant was discarded and Tween 20 was removed by centrifugation. Nanoparticles were redispersed with 5 mL of distilled water and then freeze-dried without using cryoprotectants. The same experimental procedure without OPE and Tween 20 was applied for blank gum nanoparticle production.

### 2.7. Characterization of Nanoparticles

#### 2.7.1. Nanoparticle Size and Zeta Potential

The particle size and zeta potential of nanoparticle suspensions were determined by zeta potential and a particle size meter (Nanosizer, Malvern Instruments, Worcestershire, UK) and characterized using the dynamic light scattering (DLS) technique. Before the analysis, the samples were diluted 10 times with ultrapure water and the measurement process was carried out.

#### 2.7.2. Nanoparticle Morphology

The morphological properties of dried nanoparticles were studied using a scanning electron microscope (SEM). The SEM (Zeiss EVO LS 10) has a magnification range of 20,000–50,000× and an accelerating voltage of 12 kV was used for the characterization of prepared RSG and CSG nanoparticles. All the samples were coated with gold before SEM analysis.

#### 2.7.3. Encapsulation Efficiency

The membrane filtration technique was used to determine the encapsulation efficiency of NPs by total phenolic content using a Folin–Ciocalteu method. In the fabrication of NPs, after the ultrasonication process, nanoparticle suspensions were filtered with a 10 kDa membrane filter in a stirred cell (HP4750, Sterlitech, WA, USA) at 25 °C. After the filtration, non-encapsulated OPE was collected and used for TPC analysis to determine the actual amount of OPE encapsulated in NPs. The lyophilized OPE was prepared at a rate of 0.1–1 mg/mL and a calibration curve was plotted using total phenolic content (R^2^ = 0.9843). The *EE*% of the OPE-loaded RSG and CSGNPs were calculated using the following equations:

(6) $$EE(\%)=\frac{Actual\;Amount\;of\;OPE\;Encapsulated\;in\;NPs}{Theoretical\;Amount\;of\;OPE\;Encapsulated\;in\;NPs}\times 100$$

#### 2.7.4. Thermal Properties

The thermal properties of the CSG, RSG, OPE, RSGNP, CSGNP, and OPE-loaded RSGNP and CSGNP were evaluated by differential scanning calorimetry (DSC, TA Q20, DE, USA) equipped with a thermal analysis automatic program. Samples of 5 mg were placed in an aluminum pan with a capacity of 40 µL and sealed with an aluminum lid. An empty pan was used as a reference. Conventional DSC measurements were carried out by heating the sample at a rate of 10 °C/min 30 to 400 °C under a nitrogen stream.

#### 2.7.5. Fourier-Transform Infrared Spectroscopy (FTIR)

The CSG, RSG, OPE, RSGNP, CSGNP, and OPE-loaded RSGNP and CSGNP were characterized by Fourier-transform infrared spectroscopy (FTIR) using KBr pellets in a spectrophotometer. FTIR (Bruker Tensor 27, MA, USA) characterization was performed in the transmittance mode, operating with wave numbers between 600 and 4000 cm^−1^, using 32 scans with a resolution of 4 cm^−1^.

#### 2.7.6. In Vitro Release Study

The method of Tabasi et al. (2017) [16] was modified and used to perform the release of the OPE from RSG and CSG NPs. In vitro release of OPE from NPs was determined at 37 °C for physiological pH (7.4). Phosphate buffer saline (PBS) was used to mimic the physiological pH. OPE release from RSG and CSG NPs was determined using a dialysis method. Solutions of 40 mg of RSG and CSG NPs were weighed and transferred to the dialysis bag (14 kDa) with 5 mL of distilled water. Dialysis bags were sealed on both sides and then immersed in the gastrointestinal release medium (45 mL PBS) on a shaking incubator (WiseCube Fuzzy Control System, Germany) (100 rpm) at constant temperature (37 °C). The release medium was withdrawn (0.5 mL) for analysis and replaced with a fresh medium (0.5 mL) at different time intervals (0, 30, 60, 120, 240, and 1440 min). The release of OPE was evaluated using total phenolic content spectrophotometrically (Shimadzu UV-1800, Japan). The test was carried out in triplicate.

#### 2.7.7. Determination of TPC and Antioxidant Activity of Nanoparticles

The total phenolic contents of the extract and NPs were determined according to the modified method described by Singleton et al. (1965) [34]. The total phenolic content was determined at 760 nm using a spectrophotometer (Shimadzu UV-1800, Japan) with Folin–Ciocalteu phenol reagent. The total phenolic contents of the samples were calculated as milligram gallic acid equivalent (GAE) per g dry sample. The cupric reducing antioxidant capacity (CUPRAC) analysis was carried out as previously described by Apak et al. (2004) [35]. Results were expressed as mg of Trolox equivalents per gram of dried OPE and dry weight of nanoparticles (mg TRE/g).

### 2.8. Pickering Emulsion

#### 2.8.1. Preparation Procedure

OPE-RSGNP, OPE-CSGNP, RSGNP, CSGNP, and OPE were fabricated by the same method which is explained above. The OPE-RSGNP, OPE-CSGNP, RSGNP, CSGNP (0.1% *w*/*v*) dispersions and OPE (0.01% *w*/*v*) were used to prepare the oil/water Pickering emulsions. Sunflower oil was added to nanoparticle dispersions and OPE, then ultrasonication was applied at 300 W for 20 s. The total volume of the emulsion was fixed to 50 mL. The oil volume fraction was determined φ = 0.2.

#### 2.8.2. Oxidative Stability Analysis

Oxidative stability of the Pickering emulsion samples during storage was tested using the Oxidative Tester (Velp Scientifica, Usmate, MB, Italy). A 20 g Pickering emulsion was weighed into the sample cells and the sample was distributed homogeneously. The device temperature was set to 90 °C and the oxygen pressure to 6 bar. Oxidative stability values of the samples were interpreted based on the induction period value recorded from the oxitest device.

### 2.9. Statistical Analysis

All analysis was carried out in triplicate and values expressed as mean ± standard deviation. The statistical analyses were evaluated by one-way ANOVA (Tukey’s test) using Minitab14. Statistical significance was specified as *p* 0.05.

## 3. Results and Discussion

### 3.1. Physicochemical Analysis of OPP, RSG, and CSG

The initial moisture content of the olive pomace was 38.3 ± 1.47%. The proximate compositions of OPP, RSG, and CSG are given in Table 1. OPP was comprised of a dry basis of 3.21 ± 0.03% protein, 3.39 ± 0.29% ash, 9.21 ± 0.21% fat, 3.9 ± 0.04% moisture content and 78.95 ± 0.84% carbohydrate content. RSG was composed of a dry basis of 23.01 ± 0.55% protein, 8.26 ± 0.37% ash, 0.69 ± 0.04% fat, 10.5 ± 0.5% moisture content and 57.49 ± 1.41% carbohydrate content. Koocheki et al. (2012) [21] found similar values to RSG for moisture content (12.28 ± 0.11%), higher value for ash (10 ± 0.01%) and carbohydrate content (67.92 ± 1.02%), but lower values for protein content (9.75 ± 0.9%) and fat content (trace). In their study, optimum extraction conditions of eruca sativa mucilage were found at 65.5 °C temperature, pH 4, and water:seed ratio 60:1. Karazhiyan et al. (2011) [36] studied cress seed mucilage and found similar values to RSG for protein content (22.75 ± 0.88%), but a higher value for fat content (23.4 ± 1.5%) and lower values for moisture content (5.29 ± 0.15%), ash (5.05 ± 0.11%), and carbohydrate content (43.51 ± 2.64%). As can be seen in Table 1. CSG was composed of a dry basis of 12.46 ± 0.12% protein, 9.23 ± 0.35% ash, 1.23 ± 0.14% fat, 9.5 ± 0.5% moisture content and 67.58 ± 1.11% carbohydrate content. Chia seeds were soaked in distilled water (1:30 *w*/*v*) to produce chia seed mucilage and stirred for 2 h at 25 °C [12]. de Campo et al. (2017) [12] found similar values to CSG for moisture content (11.30 ± 0.04%) and ash content (10.02 ± 1.14%), higher value for carbohydrate content as total fiber (74.04 ± 1.22%), but protein content (4.25 ± 0.00%) and fat content (0.39 ± 0.04%) were found lower than our results. Differences in the physicochemical properties of RSG and CSG are related to the extraction method used [12]. Furthermore, the extraction conditions such as water/seed ratio, temperature, and pH led to differences of physicochemical properties of RSG and CSG [36]. Variations of proximate composition of RSG and CSG also depended on rocket and chia seed variety, geographical origin, and growth conditions. These types of characteristics changed physical factors such as viscosity and thermal behavior [37].

The molecular conformation of polymer and its interactions with the aqueous phase can be predicted by determination of intrinsic viscosity. The macromolecular features of polymers can be compared by the definition of intrinsic viscosity. It is also directly related to the macromolecules’ capability to disturb flow and is indirectly related to their size and shape. Intrinsic viscosity [η] is a measure of the capability of a polymer in solution to increase the viscosity of the solution. The intrinsic viscosity values of the RSG and CSG were determined as 3.69 ± 0.4 and 19.13 ± 0.81 dL/g, respectively (Table 1). Furthermore, molecular weight of the RSG and CSG were found to be 6.82 ± 0.75 × 10^5^ Da and 2.23 ± 0.12 × 10^6^ Da, respectively. Australian chia seed gum molecular weight was 2.3 × 10^6^ and, also, intrinsic viscosity was found as 16.63 dL/g [38]. No study available can be found in the literature for intrinsic viscosity and molecular weight of the RSG. However, lower molecular weight and intrinsic viscosity of RSG were found similar for cress seed gum [39].

The phenolic composition of OPE (μg/g dried OPE) was presented in Table 2. Hydroxytyrosol (2857 μg/g), tyrosol (358.8 μg/g), and luteolin (715.6 μg/g) were identified as major phenolic compounds in the OPE by HPLC. Similar to our results, major compounds of olive pomace were reported by Skaltsounis et al. (2015), Nunes et al. (2018), and Malapert et al. (2018) [40,41,42]. Among the major phenolic compounds of OPE, hydroxytyrosol has gained attention due to having health properties such as antioxidant activity, and anti-inflammatory and antimicrobial properties [5,6].

### 3.2. Characterization of NPs

#### 3.2.1. Particle Size and Surface Charge

In this study, prepared nanoparticles showed mean particle size in the range of 304.1–318 nm for blank and OPE-loaded RSGNPs and 425.2–490 nm for blank and OPE-loaded CSGNPs, respectively (Table 3). RSGNPs had a PDI value in the range of 0.395–0.514 and CSGNPs 0.483–0.485. In this study, significant differences were observed between blank RSGNP, OPE-RSGNP, blank CSGNP, and OPE-CSGNP of the particle size (*p* 0.05). No significant differences were observed for blank and OPE-loaded CSGNPs of the PDI values. However, PDI values of OPE-loaded RSGNPs were found higher than the blank nanoparticles (*p* 0.05). Similar results were observed by Pereira et al. [43]. However, significant differences were found between blank RSGNP and CSGNP, and between OPE-RSGNP and OPE-CSGNP in the size and PDI values (*p* 0.05).

The differences in the RSG and CSG nanoparticles particle size could be related to different intrinsic viscosity and molecular weight of the RSG and CSG (Table 1). The intrinsic viscosity and molecular weight are useful parameters to control the nanoparticle size. An increase in the hydrodynamic size leads to an increase in the intrinsic viscosity [16]. A positive correlation has also been found between nanoparticle size and molecular weight [44]. As a mentioned above, the CSG had higher intrinsic viscosity and molecular weight than the RSG solutions. The higher molecular weight of gum led to higher viscosity of gum solutions [45]. The interaction of CSG and RSG molecules in the suspension with the other nanoparticle constituents could explain the obtained viscosity behavior. The viscosity behavior was formed due to the interaction between hydrocolloid suspensions and nanoparticle components. The higher molecular weight of CSG compared to the RSG led to higher particle size of OPE-CSGNP than the OPE-RSGNP (Table 3). The blank RSGNP and CSGNP tend to have a smaller size than the OPE-RSGNP and OPE-CSGNP. This result is attributed to the increased viscosity of the organic phase in the existence of encapsulated OPE. This makes it much more difficult to disperse the phases throughout the ultrasonication process and resulted in larger particles [46]. Similar results were observed by Pereira et al. (2018) [43]. Furthermore, de Campo et al. (2017) [12] found that size of the chia seed oil NPs was 205 ± 4.24 nm by using chia seed mucilage. The particle size of chitosan–oleuropein, chitosan–two different olive leaf extracts were found between the range of 250–270 nm by using chitosan as wall material [27].

As can be seen in Table 3, no significant differences were seen in the blank RSGNP and OPE-RSGNP or the blank CSGNP and OPE-loaded CSGNP. The zeta potential of the nanoparticles did not alter after the encapsulation of OPE which demonstrated that RSG and CSG are mostly located on the surface of the nanoparticles [16]. Olive leaf extract-loaded polylactic acid showed a value of −27.5 mV [25]. However, significant differences were found between blank RSGNP and CSGNP, and between OPE-RSGNP and OPE-CSGNP of the zeta potential values (*p* 0.05). This result is attributed to the stabilization effect of different kinds of gums such as RSG and CSG, which depended on composition, molecular weight, and chain lengths of gum molecules. The difference in charge density of RSG and CSG could play an important role in the stabilization effect [47]. Muhammad et al. (2020) [48] found xanthan gum and almond gum zeta potential values at −59 mV and −32 mV at % 0.5 concentration, respectively.

#### 3.2.2. Encapsulation Efficiency

As can be seen in Table 3, the EE% of olive pomace polyphenols was 82.86 ± 4.13% and 67.01 ± 4.29% in RSG and CSG NPs, respectively. The differences of the EE% value of the OPE-loaded RSGNP and CSGNP were found to be significant (*p* 0.05). These results could be explained by characteristics of the wall material composition and particle size [49]. However, the OPE-loaded RSGNP had a smaller size than the OPE-loaded CSGNP. An increase in absorption and surface volume led to higher EE% than the OPE-loaded CSGNP. The RSGNP and CSGNP covered the OPE and formed hydrogen bonding between OPE and RSGNP or CSGNP. The high EE% indicated that OPE was encapsulated in gum nanoparticles which enhanced the stability against harsh environmental conditions. Muzzalupo et al. (2020) [27] found that the EE% of the two types of olive leaf extract in chitosan nanoparticles was 94.5% and 73.1%, respectively. The values of EE% of the OPE in the literature were higher than the present study, e.g., Kesente et al. (2017) [25] for encapsulation of olive leaf extract in polylactic acid.

#### 3.2.3. Nanoparticle Morphology

**Figure 1:**
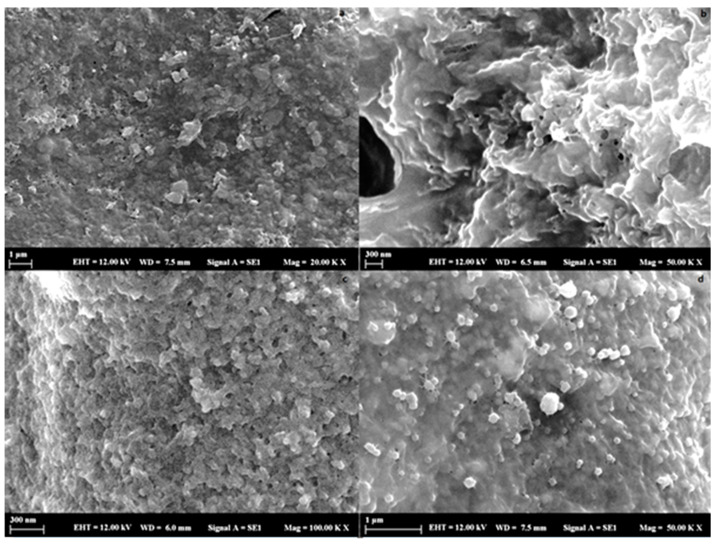
SEM images of the (**a**) RSGNP, (**b**) OPE-RSGNP, (**c**) CSGNP, and (**d**) OPE-CSGNP.

Figure 1 shows the SEM images of blank RSG and CSGNPs and OPE-RSGNP and OPE-CSGNP prepared by continuous addition of ethanol. Blank and OPE-loaded CSG-RSGNPs displayed spherical morphology with uniform size in conformance with the particle size analysis using DLS. However, irregularities in the form and roughness of the surface of the particle could be related to RSG and CSG polysaccharides. The aggregation of the particles could be explained by the fact that NPs were lyophilized before analysis and after being redispersed in a little amount of deionized water. The RSG and CSGNPs could have not been well rehydrated to prevent aggregation [50]. These results were in accordance with previous studies [47,48,51]. Furthermore, OPE-CSGNP appeared more spherical than the OPE-RSGNP. This result could be explained by the fact that zeta potential of OPE-CSGNP was higher than the OPE-RSGNP, meaning that OPE-CSGNP was more prone to aggregation than the OPE-RSGNP.

#### 3.2.4. Thermal Properties

**Figure 2:**
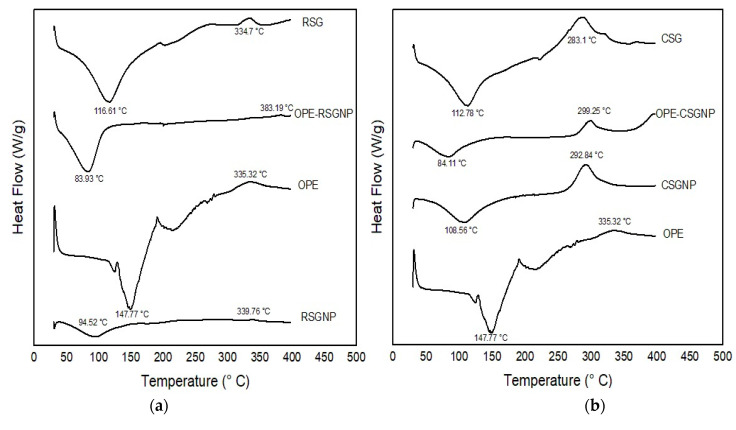
Comparison of the DSC thermograms of the (**a**) OPE, RSG, RSGNP, OPE-RSGNP, (**b**) OPE, CSG, CSGNP and OPE-CSGNP.

Figure 2 shows the DSC thermograms of blank RSG-CSGNPs, OPE-loaded RSG-CSGNPs, RSG, CSG, and OPE. The OPE presented an endothermic peak at 147.77 ± 2.55 °C and OPE also had an exothermic peak at 335.32 ± 0.53 °C. These endothermic peaks disappeared in the DSC thermogram of the OPE-loaded RSG-CSG NP, which indicated homogenous dispersion or dissolution of OPE in the polymer structure [25]. A similar result was also obtained by Kesente et al. (2017) [25] for olive leaf extract, by Doost et al. (2018) [51] for quercetin, and by Mourtzinos et al. (2007) [52] for olive leaf extract.

The RSG and CSG showed an endothermic peak at 116.61 ± 0.01 °C and 112.78 ± 0.35 °C, respectively, due to the owing free water. After the OPE-loaded RSG and CSGNPs the absence of the melting, endothermic peaks of the RSG and CSG (T_g_) in OPE-loaded RSG and CSGNPs. OPE-loaded RSG and CSGNPs showed endothermic peaks at 83.93 ± 0.01 °C and 84.11 ± 0.01 °C, respectively. The endothermic peak of OPE was not shown in the DSC thermogram of OPE-loaded RSG and CSGNPs and we also observed a shift in the T_g_ of the RSG and CSG to lower temperatures which are represented by the fact that OPE formed solid solution with RSG and CSG due to plasticizing effects [53]. These changes in peak position showed interactions and led to formation of a new structural organization of polymer and OPE [54]. A similar result was also found by Paulo and Santos (2020) [53] for tyrosol antioxidants in PCL microparticles and by Pool et al. (2012) [54] for quercetin and catechin in poly lactic-co-glycolic acid (PLGA, which is a highly biocompatible and biodegradable polymer approved by FDA) nanoparticles. The RSG and CSG had exothermic peaks at 334.7 ± 0.09 °C and 283.1 ± 0.01 °C, respectively, due to the polysaccharide structure degradation. After the OPE nanoencapsulation in RSG and CSG, exothermic peaks were formed at 383.19 ± 0.01 °C and 299.25 ± 0.09 °C, respectively. However, degradation of nanoparticles at high temperatures shows the higher thermal stability of encapsulated OPE. The OPE-RSGNP and OPE-CSGNP were more thermally stable than RSG and CSG, respectively. These results suggest that interaction formed between OPE, RSG, and CSGNPs. Furthermore, RSG and CSG could be used in the form of nanoparticles as wall material with high thermal stability, also providing good protection to OPE against degradation.

#### 3.2.5. FTIR Spectroscopy

**Figure 3:**
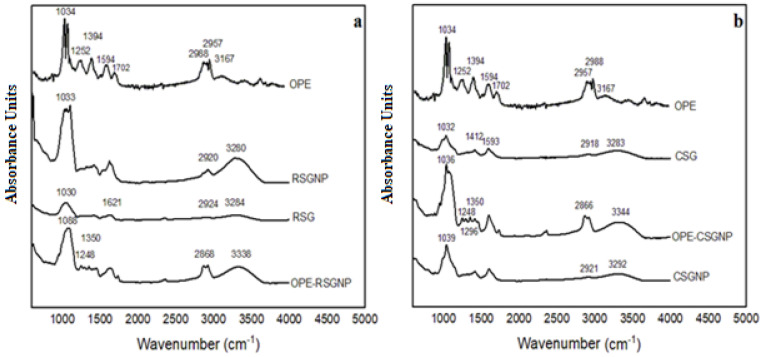
FTIR spectra of the (**a**) OPE, RSG, RSGNP, OPE-RSGNP, (**b**) OPE, CSG, CSGNP and OPE-CSGNP.

Figure 3 shows the structural properties of OPE, RSG, RSGNP, OPE-RSGNPs, and CSG, CSGNPs, OPE-CSGNPs by using FTIR spectra (600–4000 cm^−1^).

The absence of a functional group of OPE on the FTIR spectra showed the efficient incorporation of the OPE in the RSG and CSGNPs [53]. The OPE-RSG NP and OPE-CSGNP mainly displayed the absorptions of RSG and CSG which are mostly overlapped with OPE. After the encapsulation, shifted FTIR spectrum bands were observed in Table 4. The OPE-RSGNP and OPE-CSGNP also presented peaks at 1248 cm^−1^ and 1350 cm^−1^, which represented that C-O groups and deformation of OH, respectively, shifted compared to the OPE (1252.53 cm^−1^ and 1394.69 cm^−1^). The shift in wave numbers mean that interactions occurred between a phenolic compounds of OPE within the RSG and CSG, respectively.

The higher broadening of the absorption peak at 3500–2600 cm^−1^ after interaction with OPE led to increased intensity in this area, proven as O-H-O bonding, which indicated the improvement of hydrogen bonding in the OPE-loaded RSG and CSGNPs. The FTIR spectra of OPE had sharper peaks 1594 cm^−1^ and 1702 cm^−1.^ which were not observed in OPE-RSGNPs and OPE-CSGNPs. These peaks were found to overlap with the broader peaks, ranging from 1750–1500 cm^−1^. These results indicated interaction between hydroxyl groups of OPE and RSG and CSG. These results recommended that OPE was encapsulated in the nanoparticles. FTIR results revealed that RSG, CSG, and OPE had interaction during nanoparticle formation and were in good accordance with DSC results.

#### 3.2.6. In Vitro Release

**Figure 4:**
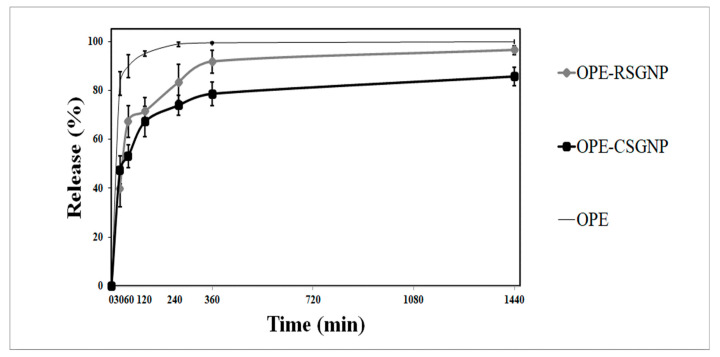
In vitro release profile of olive pomace extract (OPE) in solution and OPE-RSG, CSG NPs at small intestinal pH (7.4) for 24 h. Data are average ±standard express mean (*n* = 3).

The OPE release profiles in solution and released from the freeze-dried RSG and CSG NPs at pH 7.4 are presented in Figure 4.

During the formation of NPs, bioactive molecules are trapped both inside and on the surface of such particles. Therefore, in the first 30 min, the OPE was released from RSGNPs (39.9 ± 7.54%) and CSGNPs (47.7 ± 5.65%), which is related to the attached phenolic on the surface of the NPs. After 24 h of the incubation, it was observed that the release of OPE reached 96.45 ± 1.89% for OPE-RSGNP and 85.61 ± 3.85% for OPE-CSGNP. Initial burst release occurred probably due to the OPE on the surface, followed by an accumulative release [27,55].

The OPE was covered by RSG and CSGNPs and thus prevented OPE from releasing rapidly. OPE-loaded RSGNPs had a lower particle size (Table 3) than the OPE-loaded CSGNPs. This result could be explained as complexes with smaller particle size may have a bigger surface to volume ratio and buffer penetration in the nanoparticles, resulting in the quick release of encapsulated compounds adsorbed on the surface [56].

The in vitro release study showed that the RSG and CSG systems could play a significant role in preventing the discharge of the OPE and, consequently, controlling the release of polyphenol in the physiological pH. Free OPE was suspended in the release medium to determine its stability in in vitro release conditions. The OPE was suspended in the release medium at 82.79 ± 4.54% and after 4 h the OPE reached to 98.9 ± 1.09% It could be stated that the encapsulation of OPE in RSG and CSGNPs led to the delay of the OPE release in physiological pH up to 24 h.

#### 3.2.7. Antioxidant Activity of the Nanoparticles

The antioxidant activities of blank RSGNP, CSGNP, OPE, and OPE-loaded RSG, CSG nanoparticles are given in Table 5. As can be seen in Table 5, the OPE had higher total phenolic content (44.24 mg GAE/g) and cupric reducing antioxidant capacity (254.56 mg TE/g) than the other samples. As seen in Table 5, after loading RSGNPs and CSGNPs with OPE, the TPC values of RSGNPs and CSGNPs increased by 10.89 mg/g and 9.27 mg/g, respectively. Similarly, the antioxidant activity of both nanoparticles increased more than 50%. These results showed that nanoparticles can encapsulate phenolic compounds in OPE and therefore TPC and antioxidant activity of nanoparticles increased. The increases in TPC and antioxidant values in RSGNPs with OPE loading are higher than those of CSGNPs. The higher increase in TPC and antioxidant value of RSGNPs could be explained by the higher encapsulation efficiency value of RSGNPs (82.86%) than CSGNPs (67.01%).

The type of wall materials can influence total phenolic content and the antioxidant activity of the nanoparticles [57]. Therefore, TPC and antioxidant values of the RSGNPs and CSGNPs before OPE loading were also measured to determine the increase in the antioxidant activities of nanoparticles with OPE loading. Natural polysaccharides are not always found singly, as they are conjugated with other constituents such as phenolic compounds, amino acids, protein, lipids, and nucleic acid residues. Furthermore, the content of protein in polysaccharide improved direct scavenging activity on superoxide and hydroxyl radicals. Polysaccharides containing functional groups such as OH, -SH, -COOH, -PO_3_H_2_, -C=O, -NR_2_, -S-, and -O- are in favor of chelating ability. These functional groups were shown in FTIR spectrum of RSG and CSG and have chelating and antioxidant activities. In addition, molecular weights of polysaccharides are associated with antioxidant activity. Polysaccharides with low molecular weight have more hydroxyl groups to accept and eliminate the free radicals and show higher antioxidant activity [58]. The RSG had lower molecular weight than CSG and showed higher antioxidant activity. Malsawmtluangi et al. (2014) [59] found that increasing polysaccharide concentration led to increase in antioxidant activity. Furthermore, the blank RSGNP and CSGNP of the TPC and CUPRAC showed good correlation. A similar result was reported by Shaygannia et al. (2020) and Ahmed et al. (2010) [57,60]. In conclusion, a significant increase was observed in TPC and antioxidant values of both nanoparticles with OPE loading. This result shows that both nanoparticles can be used successfully in the encapsulation of OPE phenolics.

#### 3.2.8. Oxidative Stability of the Pickering Emulsions

Table 5 presents the IP values of the Pickering emulsions. As can be seen in Table 5, the OPE oil in water (O/W) Pickering emulsion had lower IP value (h) (2.51 ± 0.02 h) than the other samples. Table 5 shows the oxidative stability values of emulsions prepared using nanoparticles. As can be seen, a significant difference was observed between the IP values of the samples. The IP values of all nanoparticle samples were higher than the IP values of the emulsion prepared with OPE only. The IP values of the emulsion prepared OPE-RSGNPs (4.39 ± 0.11 h) and OPE-CSGNPs (3.23 ± 0.07 h) nanoparticles were higher than those of RSGNPs (4.10 ± 0.08 h) and CSGNPs (2.59 ± 0.07 h). These results were in agreement with the antioxidant results of nanoparticles. The higher IP value of the OPE-loaded nanoparticles can be explained by the more effective scavenging of free radicals with the controlled release of the loaded phenolic compounds. The higher IP value of OPE-RSGNPs compared to OPE-CSGNPs can be explained by the higher phenolic compound loading capacity and higher antioxidant activities of OPE-RSGNPs due to the higher encapsulation efficiency.

The results show that OPE-loaded nanoparticles slow down the oxidation of the Pickering emulsions. These increases could be explained by the localization of OPE phenolic compounds at the oil in water interface of the Pickering emulsions. The interaction of OPE phenolic compounds with other antioxidant compounds could have improved antioxidant activity and led to higher IP values [61,62]. The oxidative stability results were consistent with antioxidant activity of NPs. In Table 5, OPE had higher antioxidant activity than the NPs. However, nonencapsulated OPE had lower IP value than the NPs due to the quick degradation of OPE phenolic compounds at high temperatures, such as 90 °C. Furthermore, OPE-loaded RSG and CSGNPs had higher IP values, indicating that nanoencapsulated OPE could have increased oxidative stability of Pickering emulsions due to the prevention of degradation of OPE phenolic compounds by using natural gums as wall materials for nanoencapsulation.

## 4. Conclusions

RSG and CSGNPs were fabricated to determine the interaction and protective capacities of the RSG and CSG as wall materials for OPE. The OPE-loaded RSG and CSGNPs were confirmed to have high encapsulation efficiency by FTIR characteristic bands and SEM images. The nanoencapsulated OPE increased oxidative stability of Pickering emulsions due to preventing the degradation of OPE phenolic compounds by using natural gums as wall material for nanoencapsulation. The results showed that the RSG and CSGNPs could be used as wall materials for delivery of bioactive compounds such as OPE phenolic nanoencapsulation for food industries, and that RSG and CSG could be alternatives to substitute synthetic polymers. Further studies are needed to improve the release behavior and stability of nanoparticles under the different process conditions.

## Figures and Tables

**Table 1:** Physicochemical properties of olive pomace, rocket seed and chia seed gum.

Sample	Olive Pomace Powder	Rocket Seed Gum	Chia Seed Gum
pH	5.06 ± 0.1	6.24 ± 0.14	6.85 ± 0.15
Carbonhydrates (% *w*/*w*)	78.95 ± 0.84	57.49 ± 1.41	67.58 ± 1.11
Fat (% *w*/*w*)	9.21 ± 0.21	0.69 ± 0.04	1.23 ± 0.14
Ash (% *w*/*w*)	3.39 ± 0.29	8.26 ± 0.37	9.23 ± 0.35
Moisture (% *w*/*w*)	3.9 ± 0.04	10.5 ± 0.5	9.5 ± 0.5
Protein (% *w*/*w*)	4.55 ± 0.30	23.01 ± 0.55	12.46 ± 0.12
Instrinct Viscosity (dL/g)	n.d.	3.69 ± 0.4	19.13 ± 0.81
Molecular Weight (Da)	n.d.	6.82 ± 0.75 × 10^5^	2.23 ± 0.12 × 10^6^

n.d.: not detected.

**Table 2:** Phenolic compounds of OPE.

Phenolic Compounds	Retention Time (min)	Concentration (µg/g Dried OPE)
Gallic acid	5.867	314.4
Protocatechuic acid	8.864	60.4
Hydroxytyrosol	9.3	2857.0
Catechin	12.360	48.8
p-Hydroxybenzoic acid	13.429	15.6
Tyrosol	14.3	358.8
Syringic acid	14.919	16.4
Elagic acid	20.495	134.4
m-Coumaric acid	22.623	15.2
o-Coumaric acid	25.082	2.8
Myricetin	26.950	348.4
Quercetin	33.083	217.2
Kaempferol	36.162	68.8
Luteolin	74.8	715.6

**Table 3:** Particle size, zeta potential and encapsulation efficiency of nanoparticle suspensions.

Sample	Particle Size (nm)	PDI	Zeta Potential (mV)	EE (%)
RSGNP	304.1 ± 4.49 ^D^	0.395 ± 0.01 ^C^	−23.1 ± 0.85 ^A^	
OPE-RSGNP	318 ± 3.11 ^C^	0.514 ± 0.06 ^A^	−22.6 ± 1.23 ^A^	82.86 ± 4.13 ^A^
CSGNP	425.26 ± 6.49 ^B^	0.485 ± 0.08 ^B^	−28.1 ± 0.95 ^B^	
OPE-CSGNP	490 ± 8.67 ^A^	0.483 ± 0.10 ^B^	−29.9 ± 2.57 ^B^	67.01 ± 4.29 ^B^

The mean values with the same letter do not differ significantly (*p* 0.05) and different letters differ significantly (*p* 0.05) in the same column.

**Table 4:** FTIR spectrum bands of NPs.

FTIR Spectrum of	Compared with FTIR Bands of	Band (cm^−1^) Observed	Observed Shift after Encapsulation
OPE-RSGNP	OPE	2988 and 2957 cm^−1^ C-H stretching, especially asymmetric and symmetric vibration	2868 cm^−1^
		3167 cm^−1^ corresponding to O-H hydroxyl group	3338 cm^−1^
		1594, 1702 cm^−1^ corresponding to C=O stretching of the carbonyl groups	-
		1104, 1074, 1034 cm^−1^ attributed to the C-O stretching of the ester groups	1088 cm^−1^
		1394 and 1252 cm^−1^ corresponding to stretching of C-O groups and deformation of O-H, respectively	1350 cm^−1^ 1248 cm^−1^
	RSGNP	3280 cm^−1^ corresponding to O-H hydroxyl group	3338 cm^−1^
		2920 cm^−1^ the bond between the C-H groups, namely the CH_2_ stretch	2868 cm^−1^
		1033 cm^−1^ shows the C-O-C bonds	1088 cm^−1^
OPE-CSGNP	OPE	2988 and 2957 cm^−1^ C-H stretching especially asymmetric and symmetric vibration	2866 cm^−1^
		3167 cm^−1^ corresponding to O-H hydroxyl group	3344 cm^−1^
		1594, 1702 cm^−1^ corresponding to C=O stretching of the carbonyl groups	-
		1104, 1074, 1034 cm^−1^ attributed to the C-O stretching of the ester groups	1071 cm^−1^ 1036 cm^−1^
		1394 and 1252 cm^−1^ corresponding to stretching of C-O groups and deformation of O-H, respectively	1350 cm^−1^ 1296 cm^−1^ 1248 cm^−1^
	CSGNP	3292 cm^−1^ corresponding to O-H hydroxyl group	3344 cm^−1^
		2921 cm^−1^ the bond between the C-H groups, namely the CH_2_ stretch	2866 cm^−1^
		1039 shows the C-O-C bonds	1071 cm^−1^ 1036 cm^−1^

**Table 5:** TPC and antioxidant activity of nanoparticles and oxidative stability of Pickering emulsions prepared by nanoparticles.

Sample	Bioactive Properties of OPE and Gum Nanoparticles		Oxidative Stability of Pickering Emulsions
	TPC (mg GAE/g)	CUPRAC (mg TE/ g)	IP Value of
OPE	44.24 ± 0.22	254.56 ± 1.70	2.51 ± 0.02
OPE-RSGNP	27.92 ± 1.01 ^A^	235.28 ± 0.36 ^A^	4.39 ± 0.11 ^A^
RSGNP	17.08 ± 0.5 ^B^	148.49 ± 7.45 ^B^	4.10 ± 0.08 ^B^
OPE-CSGNP	19.52 ± 0.33 ^a^	212.07 ± 50 ^a^	3.23 ± 0.07 ^a^
CSGNP	10.25 ± 1.13 ^b^	135.28 ± 0.36 ^b^	2.59 ± 0.07 ^b^

The mean values with the same letter do not differ significantly (*p* 0.05) and different letters differ significantly (*p* 0.05) in the same line. A and B signify for OPE-RSGNP and RSGNP, a and b signify for OPE-CSGNP and CSGNP.

## Data Availability

The data presented in this study are available on request from the corresponding author.
